# Achieving brain clearance and preventing neurodegenerative diseases—A glymphatic perspective

**DOI:** 10.1177/0271678X20982388

**Published:** 2021-01-18

**Authors:** Tekla Maria Kylkilahti, Eline Berends, Marta Ramos, Nagesh C Shanbhag, Johannes Töger, Karin Markenroth Bloch, Iben Lundgaard

**Affiliations:** 1 Department of Experimental Medical Science, Lund University, Lund, Sweden; 2 Wallenberg Centre for Molecular Medicine, Lund University, Lund, Sweden; 3 Diagnostic Radiology, Department of Clinical Sciences, Lund University and Skane University Hospital Lund, Lund, Sweden; 4 Lund University Bioimaging Center, Lund University, Lund, Sweden

**Keywords:** Glymphatic system, cerebrospinal fluid, 7T MRI, brain clearance, neurodegeneration

## Abstract

Age-related neurodegenerative diseases are a growing burden to society, and many are sporadic, meaning that the environment, diet and lifestyle play significant roles. Cerebrospinal fluid (CSF)-mediated clearing of brain waste products via perivascular pathways, named the glymphatic system, is receiving increasing interest, as it offers unexplored perspectives on understanding neurodegenerative diseases. The glymphatic system is involved in clearance of metabolic by-products such as amyloid-β from the brain, and its function is believed to lower the risk of developing some of the most common neurodegenerative diseases. Here, we present magnetic resonance imaging (MRI) data on the heart cycle’s control of CSF flow in humans which corroborates findings from animal studies. We also review the importance of sleep, diet, vascular health for glymphatic clearance and find that these factors are also known players in brain longevity.

## Introduction

As life expectancy has increased in many countries, aging-related neurodegenerative disorders such as dementia or movement disorders are growing in prevalence. Alzheimer’s disease (AD) and Parkinson’s disease (PD) are the most common neurodegenerative diseases. Currently, no cure is available although epidemiological studies suggest that the risk of developing neurodegenerative diseases can be modulated by lifestyle-related factors, suggesting that some cases could be prevented. The toxic accumulation, misfolding or mis-localisation of proteins leading to neuronal loss, i.e. proteinopathies, are key pathological features of age-related neurodegenerative diseases.^1^ Breakdown or removal of the proteins which are susceptible to form toxic aggregates is essential to prevent development of pathology.^2,3^ Many of these proteins, such as AD associated amyloid-β and tau and PD associated α-synuclein, are found in the cerebrospinal fluid (CSF). This raised the question of the significance of CSF for clearing toxic metabolites from the brain, and in 2012, the glial-lymphatic (“glymphatic”) system, that describes a mechanism for brain clearance via a perivascular (also referred to as paravascular) CSF flow pathway was characterised.^4^ Indeed, the glymphatic system plays a role in clearance of amyloid-β,^4–7^ tau,^8,9^ and α-synuclein.^10^

**Figure 1.**
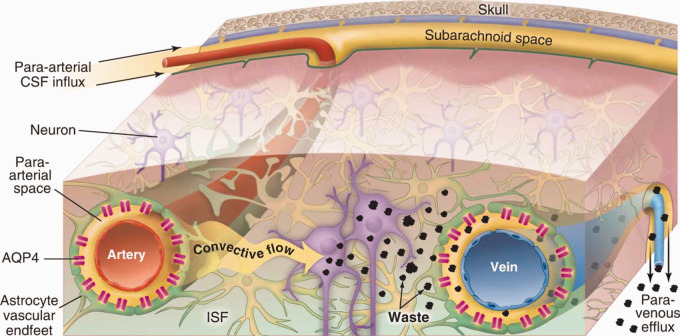
CSF-ISF exchange facilitates waste clearance from the brain. The glymphatic system describes a waste clearance pathway in the brain that relies on interchange of cerebrospinal fluid (CSF) and interstitial fluid (ISF). CSF flows into the parenchyma from the periarterial spaces surrounding cerebral vasculature, and this interchange is facilitated by aquaporin-4 water channels. Fluid exits the parenchyma via the perivenous spaces, and can remove various waste products, such as amyloid-β. (Reprinted with permission from Nedergaard^13^).

The glymphatic brain clearance mechanism relies on interchange of CSF and interstitial fluid (ISF) that allows waste to be transferred to the CSF and transported out of the brain.^4,11^ The system was named the glia-lymphatic or “glymphatic” system upon its discovery in 2012 as astrocyte end feet are a main structural component of the fluid exchange pathway.^4^ CSF is predominantly produced in the choroid plexus in the 3rd and lateral ventricles, and it is circulated from the ventricles to the subarachnoid space surrounding the brain primarily by arterial pulsations.^12^ The subarachnoid space is continuous with the periarterial spaces of the pial vessels, from which the CSF enters the brain parenchyma, where it facilitates the clearance of solutes, although the efflux routes are less described (Figure 1).^4,13^ The interchange of CSF and ISF is dependent on aquaporin 4 (AQP4) water channels on astrocyte endfeet that enwrap the cerebral vasculature.^4,6^ Changes in AQP4 expression or polarisation – referring to the differential distribution of AQP4 in the endfeet versus rest of the cell – are associated with disturbances in glymphatic function.^14,15^ In line with the observation that the glymphatic system can clear amyloid-β, decreased glymphatic function caused by deletion of the *Aqp4* gene in an animal model of Alzheimer’s disease leads to increased accumulation of amyloid-β^2^ and tau.^16^ Abnormalities in AQP4 polarisation are also seen in Alzheimer’s patients, which provides some evidence that glymphatic function might also play a role in Alzheimer’s disease in humans.^17^

Research in animals has shown that glymphatic activity can be modulated by a number of dietary, behavioural, and physiological changes which can be affected by lifestyle in humans. Here we carried out a 7 Tesla magnetic resonance (7 T MR) visualisation of the fact that the CSF flow in the aqueduct is correlated with the heart cycle in humans, and we discuss these results in the context of diet, vascular function and the glymphatic system. Additionally, we review the interplay between lifestyle and brain health.

## Material and methods

### MR image acquisition

We used ultra-high field 7 T MR (Achieva, Philips, Best, The Netherlands) with a dual-channel transmit/32-channel receive head coil (NOVA Medical, Wilmington, MA, USA) to perform 2 D PC MR velocity and flow quantification in 9 healthy volunteers, as previously described.^18^ The CSF flow measurement was acquired in the cerebral aqueduct, with an image resolution of 0.3x0.3 mm^2^ and a 5 mm thick imaging slice. A high resolution is crucial to avoid underestimation of flow in the narrow aqueduct. The slice was positioned perpendicularly to the aqueduct after identifying it on a 3 D T1 weighted MR image. The 2 D PC MR acquisition was synchronised with the heart beat using ECG detection. In one subject, we additionally acquired 4 D PC flow data covering the whole aqueduct. The technique can be used to assess changes is the flow in longitudinal studies of disease or after physiological challenges.

All volunteers gave informed consent and the experiment had ethical approval and followed the ethical guidelines from the Swedish Regional Ethical Review Board (Etikprövningsmyndigheten No. 2012-428).

### Analysis

CSF flow data were analysed using Segment v2.2 R7052 (http://segment.heiberg.se) by manually delineating the cerebral aqueduct in the cardiac phase with maximal flow and copying that delineation to all time frames. Statistical analysis was performed with Prism v8.2.1 (GraphPad, La Jolla, Ca, USA). To study the CSF flow patterns over the heart cycle, the RR-interval, as recorded by 7 T, was normalised to 60 bpm in all subjects.

## Cerebrovascular function drives macroscopic CSF flow and perivascular CSF-ISF exchange

**Figure 2.**
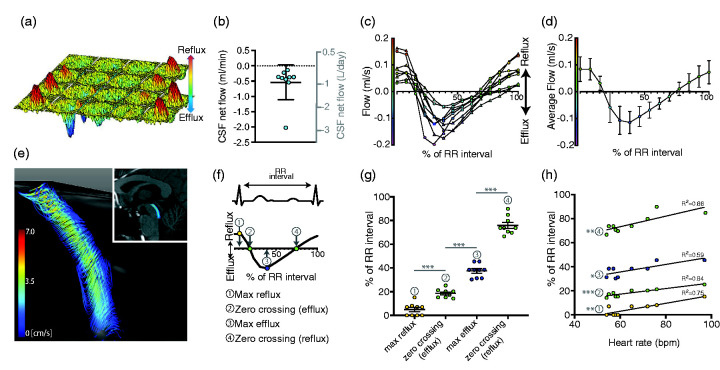
CSF movement in the cerebral aqueduct is driven by the cardiac cycle. CSF flow in the cerebral aqueduct was measured with 7 T MR synchronized to the cardiac cycle with the temporal axis starting at the R-wave (onset of systole). (a) Representative surface plot of the pulsatile, in-and-out flow of CSF in the aqueduct. Reflux and efflux refer to flow of CSF towards the 3rd and 4th ventricle, respectively. (b) Net CSF flow ranged from –0.14 to –2.02 ml/min, or –200 to –2910ml/day, minus indicating efflux (n = 9). (c) CSF flow in the aqueduct from nine healthy subjects across a heart cycle normalized to 60 bpm, measured with 7 T MR. (d) Average CSF flow data for nine healthy subjects. Error bars show standard deviation. (e) 4 D flow review reconstruction of the CSF flow patterns in the cerebral aqueduct, extending from the base of the 3rd ventricle to the 4th ventricle, shown superimposed on the sagittal view of the brain. (f) The time points along the RR interval for (g) and (h), corresponding to peak reflux (1), zero crossing (2), the time of peak efflux (3) and zero crossing (4). (g) The temporal distribution of characteristics of the flow curve corresponds to specific phases of the cardiac cycle. ***p < 0.001, 2-way ANOVA. (h) Timing of events in relation to RR-interval correlates moderately (two later events, 3 and 4) and strongly (two early events, 1 and 2) with the heart rate. *p < 0.05, **p < 0.01, ***p < 0.001, Pearson Correlation. Previously unpublished data.

Pulsations of the brain caused by the inflow of blood during the cardiac systole are easily observable during craniotomies. This drives pulsations of CSF in the ventricles and is largely responsible for the circulation of CSF from the site of production to the subarachnoid space.^12^ Further downstream are the perivascular spaces of pial vessels, where CSF and ISF exchange takes place. Disturbances in macroscopic CSF circulation are observed in diseases such as idiopathic normal pressure hydrocephalus^19,20^ and aqueductal obstructions,^21^ where flow measurement with MRI are used clinically as a diagnostic tool, but there are also indications of changes in Alzheimer’s disease, dementia^20,22^ and multiple sclerosis.^23^ Assessing macroscopic CSF flow might therefore have diagnostic value or be indicative of dysfunction in the downstream compartments such as the perivascular spaces essential for brain clearance. To further investigate the dynamics of the CSF pulsations in vivo, we quantified the velocity and flow of CSF in the cerebral aqueduct throughout the cardiac cycle non-invasively using 2 dimensional phase contrast MR (2 D PC MR).^24,25^ As CSF is primarily produced in the choroid plexus of the lateral, third and fourth ventricles, the net CSF flow in the aqueduct between the third and fourth ventricle can be used as a proxy for the amount of CSF added or re-circulated to the downstream compartments (Figure 2(a) to (e)). The challenge of aqueductal measurements is that due to its small diameter, sufficient accuracy can only be obtained at a high in-plane image resolution. Previously, PC MRI of the aqueduct has been carried out primarily using lower resolution 1.5 T and 3 T scanners and has been used e.g. to study treatment responses in idiopathic normal pressure hydrocephalus.^19,26,27^ By using ultra-high field MRI (7 T) we obtained a higher image resolution which is particularly beneficial for accuracy when measuring flow in small regions such as the aqueduct.^18^ We synchronised the 2 D PC MRI with the heartbeat using ECG detection to obtain the flow curve over the cardiac cycle. CSF flow measurements were acquired at 7 T with a resolution of 0.3x0.3 mm^2^ and a 5 mm thick imaging slice positioned perpendicular to the aqueduct. The data show that the CSF pulsatility in the aqueduct is highly correlated with discrete phases of the cardiac cycle. Consistently for all healthy subjects, the CSF efflux to the fourth ventricle peaks during systole while reflux back into the third ventricle peaks during the late diastolic phase (Figure 2(c) to g, 2(f) to (h)). Although in synchrony with the heart cycle, aqueductal movement of CSF shows a delay corresponding to the pulse wave delay in humans. This suggests that the cardiac cycle drives macroscopic CSF flow. Aqueductal CSF flow is also affected by other physiological variables such as respiration,^28,29^ however, by sampling over several minutes the effects of respiration were averaged out in our data. Despite the highly pulsatile back-and-forth nature of CSF flow, there was a net CSF efflux in the aqueduct in line with CSF production rates reported in the literature,^12^ suggesting that MRI of aqueductal flow can be indicative of CSF production (Figure 2(b)).

**Figure 3.**
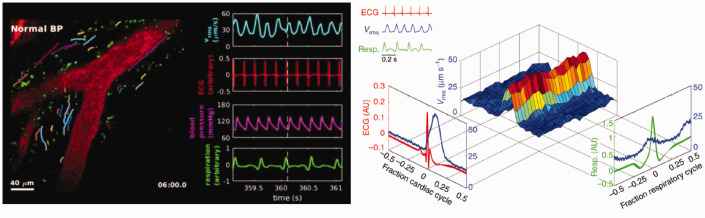
Glymphatic flow is driven by the cardiac cycle. Left: Two-photon imaging in live mice through a cranial window after cisterna magna injection of fluorescent microspheres. Graphs show the velocity (measured as the root-mean-square) in blue, ECG in red, blood pressure in pink, and respiration in green. Right: Surface plot visualising the how the peak traced particle velocity (V_rms_, in blue) in the perivascular space closely follows the R peak of the electrocardiogram (in red), and to lesser extent ventilation pattern (in green). This suggest that the cardiac cycle is a powerful driver of perivascular flow. (Adapted from Mestre et al (2018), Nature Communications, Figure 3 and Supplementary movie 3. Reprinted under Creative Commons CC BY 4.0 licence).

These data are in line with a particle tracking study which showed that the CSF movement at the level of the perivascular spaces is driven by cardiac pulsations,^30^ suggesting arterial pulsations are important for CSF circulation in different anatomical regions. In this comprehensive study, velocimetry of over 20,000 fluorescent microspheres in conjunction with cardiac and respiratory monitoring in mice revealed that also the CSF flow along the perivascular spaces is pulsatile in synchrony with the cardiac cycle (Figure 3). Forward movement of the particles followed the pulsations of the cardiac systole in a nearly discrete manner, with some backwards movement in the diastolic phase, thereby following dilation and contraction. Even experimental increase of the vascular pulsatility using a beta-adrenergic agonist enhanced the CSF-ISF exchange.^31^ Cardiac-driven motion of the vessel wall thus drives the bulk flow of CSF in direction of the blood flow, thereby supporting the hypothesis of the perivascular pumping phenomenon.^30^ Vasomotion of vessels in general, i.e. spontaneous constriction and dilation of a vessel independent of the pulsatile blood flow, also regulated perivascular fluid dynamics, as induction of vasomotion by functional activation led to enhanced perivascular clearance of fluorescent tracer.^32^ Interestingly, the impairment of the evoked vasomotion and clearance rates in aged AD mice was linked to the loss of vascular smooth muscle cells, thus indicating that AD pathology aggravates decline of glymphatic function with age.^15^ The perivascular pumping phenomenon and vasomotion thus appear to be drivers of glymphatic function, which highlights the importance of vascular health for brain clearance, as the cardiac cycle is a driver of CSF flow at both perivascular and macroscopic level.

## Physical exercise improves glymphatic function in rodents

Exercise has beneficial effects on brain health; it reduces risk of neurological disorders and can improve cognitive function.^33^ Exercise during midlife reduces risks for developing dementia and AD decades later.^34^ Some of these beneficial effects might be supported by exercise-enhanced brain clearance, as two groups have shown that voluntary exercise can improve glymphatic function in rodents.^35,36^ In terms of glymphatic pathway activation, voluntary running significantly improved the CSF-ISF exchange and CSF efflux via drainage into the deep cervical lymph nodes when compared with control groups.^35,36^ Exercise also resulted in reduced amyloid-β levels, glia cell immunoreactivity and increased AQP4 polarisation as well as improved cognition compared to the sedentary group.^35^ Interestingly, the beneficial effects of exercise on glymphatic function are not seen acutely during exercise,^36^ and thus likely not mediated by the increased pulse rate and cardiac output. As low noradrenaline levels during sleep or as a result of pharmacological intervention are correlated with increased glymphatic function,^37^ exercise induced changes on adrenaline and noradrenaline signalling may explain the acute reduction in glymphatic influx during exercise.

Additionally, exercise exerts beneficial effects on vascular health and increases vascular compliance.^38^ Cerebral blood flow (CBF) is a driver of glymphatic function,^30,31^ and there is a growing body of evidence suggesting that dysregulation of CBF plays a role in both vascular and neurodegenerative dementias.^39^ Restriction of CBF reduces arterial wall pulsatility, thus reducing CSF-ISF exchange in the brain, suggesting a functional connection between cerebral haemodynamics and the glymphatic system.^31^ Furthermore, the potential for enhancement of perivascular flow by improving vascular compliance would be in line with the data showing that hypertension reduces both vascular compliance and glymphatic function.^30,40^ The pulse wave velocity (PWV) is increased in peripheral arteries as vascular compliance is reduced.^41^ Increased PWV can be seen in patients with mild cognitive impairment (MCI) or dementia,^41^ which suggests a connection between compliance and cognitive function. The link between exercise, vascular compliance and the glymphatic system is interesting, as it might clarify the link between vascular diseases and neurodegeneration. This points to a dual role for the circulatory system in terms of maintaining CNS health, as it drives perivascular fluid movement in addition to blood supply itself, in line with blood flow being a marker for a healthy brain. Taken together, the evidence presented above converge on the suggestion that modulation of CBF or cerebral vasomotion can enhance perivascular clearance and improve CSF-ISF exchange, under similar conditions that are known to enhance cognition in humans. Cerebrovascular insufficiencies as encountered during aging or vascular diseases of the brain could perhaps result in an inadequate waste clearance due to impaired vasomotion, leading or contributing to neurodegenerative diseases such as AD.

## Neurodegeneration and sleep are connected in a bidirectional manner

Disruptions of circadian rhythm and fragmented sleep are believed to be connected to the development of PD and AD in a bidirectional manner, suggesting sleep disturbances are both a symptom and a potential contributor to these diseases.^42^ Glymphatic function emerged as a potential link between poor sleep and neurodegenerative diseases, as glymphatic function is almost exclusively active in the sleep stage,^5^ assuming that reduced interstitial clearing of e.g. amyloid-β plays a role in developing PD and AD. Owing to the apparent importance of sleep on brain clearance, we here summarise what is known on the relation between sleep and brain health.

Sleep is necessary for a wealth of biological functions, and short or disrupted sleep have both acute and chronic adverse effects on health. Chronic sleep deprivation, sleep disruption and even shift work are connected to an increased prevalence of pathologies, including the metabolic syndrome, cardiovascular disease^43^ and some cancers.^44^ Sleep deprivation has been connected to cognitive dysfunction.^45,46^ Interestingly, sleep disturbances and circadian dysfunction are common in AD and PD patients,^47,48^ and even observed in mouse models of AD.^49^ Huntington’s disease and Lewy body dementia are strongly associated with changing sleep quality, while frontotemporal dementia is associated with sleeping problems.^50^

Post mortem tissue from AD patients with daytime sleepiness showed loss of hypocretin (orexin),^51^ which is one of the main regulators of the sleep/wake cycle.^52^ Some studies report increased hypocretin levels in AD and MCI patients^53^ which is linked to night time wakefulness.^54^ Interestingly, the hypocretin levels also correlated with increased levels of amyloid-β.^54^ A small clinical study suggested that autoimmunity to AQP4 may cause hypothalamic lesions and lead to disruption of hypocretin signalling in Multiple Sclerosis patients.^55^ Building upon the hypothesis that AQP4 expression can influence sleep, it was suggested that genetic variation in AQP4 leads to hypothalamic dysfunction, thus providing a possible connection between sleep problems and increased amyloid burden.^56^ Another sleep-regulating neuropeptide, melatonin, is also reduced in AD patients at night and increased during the day due to damage to the suprachiasmatic nucleus.^57^ In PD, it is believed that the damage to the dopaminergic neurons causes disruptions in sleep, and in later stages of PD α-synuclein aggregates in sleep-associated areas thus exacerbating sleeping disorders.^58^ Furthermore, neurodegeneration seems to lead to disruption of pathways needed for glymphatic function and regulation of sleep. AD patients show a higher degree of perivascular enlargement compared to healthy controls.^59^ Interestingly, enlargement of perivascular spaces in itself is connected to poor sleep quality.^60,61^ A population study found that enlarged perivascular spaces and sleep disruptions are linked, and it was therefore hypothesised that sleep deficiency may lead to structural changes in the perivascular spaces.^61^ Another study found a connection between the quantity of enlarged perivascular spaces and decreased sleep time.^62^ Taken together, these data suggest that disturbed sleep and circadian dysfunctions as a consequence of AD and PD are at least partly due to direct cell damage to the brain regions governing sleep and wakefulness, and sleep disturbances may further aggravate disease progression via accumulation of metabolic by-products.

In fact, increased daytime napping – symptomatic of circadian dysregulation – correlates with amyloid deposits.^63^ Sleep disturbances start already in the preclinical stages, i.e. cognitively normal individuals with poor sleep quality have significantly higher amyloid levels, suggesting that sleep disturbances may be a risk factor for AD.^63^ In line with the hypothesis that disturbed sleep patterns lead to reduced brain clearance and proteinopathies, there is a strong inverse correlation observed between hours slept and amyloid-β levels in the cortex of humans.^64^ Both animal and clinical studies suggest that deep sleep in particular is vital for brain clearance, as slow (delta) waves that occur during stage three of NREM sleep are associated with high levels of glymphatic influx^65^ and amyloid-β clearance.^66^ The connection between delta waves and increased glymphatic function can also be seen in anaesthetised mice, where anaesthetics that promote delta waves more have the highest influx in the entire brain.^5,67,68^

In addition to the state-dependent changes in awake versus sleep state, diurnal fluctuations in neurodegenerative-associated protein levels in the ISF have been observed already before the glymphatic system was conceptualized.^69^ In rodents, the levels of amyloid-β^69–71^ and tau^70^ are relatively high during wakefulness and low during sleep. In a natural sleep cycle in humans, levels of amyloid-β_40_ in CSF peak during the evening and is lowest in the morning.^69^ The circadian fluctuations in both the glymphatic activity and levels of waste products point to the importance of the circadian rhythm as a regulator of brain clearance.^72^ However, since the sleep-induced fluctuations can be manipulated with sleep deprivation^70^ or anaesthetics,^5^ it appears that the state dependent changes are a more powerful regulator of glymphatic function than the circadian timepoint alone.^11,72^ This conclusion is supported by studies that use anaesthetics to activate the glymphatic system. This would suggest that not the circadian time point, but presumably the sleep or a sleep-like brain state is a main regulator of glymphatic activity.

Sleeping posture is a proposed risk factor for neurodegeneration.^73,74^ A small clinical study in humans has shown that neurodegenerative disease patients tend to spend more time sleeping in supine position (on the back) than healthy controls, who instead prefer lateral positions (on the side).^74^ In rats, CSF flow is optimal when positioned in lateral and supine positions, while correspondingly reduced in the prone position.^7^ Head position also has an effect on amyloid-β clearance rate.^7^ Thus, the beneficial effect of sleep is affected by something as simple as the position of the head, further indicating a role for fluid dynamics as a player in brain clearance.

## Glymphatic function is increased during sleep and impaired by sleep disturbances

In healthy humans, studies have shown that one night of sleep deprivation leads to increases in amyloid-β plaques in the hippocampus,^64^ amyloid-β_42_,^75^ ISF tau^70^ and CSF tau and α-synuclein.^70^ Similar to the effects seen after acute sleep deprivation, chronic insomnia patients had higher CSF levels of amyloid-β_42_ than normally sleeping individuals.^76^ There is also a correlation between amyloid-β_42_ levels and sleep quality as well as time since onset of insomnia^76^ suggesting detrimental effects of insomnia display dose-dependent patterns.

These findings can be recapitulated in animal models. Increase in ISF amyloid-β levels can be seen as an effect of a single night of sleep deprivation and when chronic, sleep deprivation leads to increased amyloid-β plaque burden leads in an AD mouse model.^69^ In AD mice, chronic sleep deprivation also resulted in an increase in insoluble amyloid-β_42_, both acutely but interestingly even after sleep discovery.^45^ Likewise, tau pathology in the parenchyma is exacerbated in a mouse model of AD following chronic sleep deprivation.^77,78^ In terms of glymphatic function, sleep deprivation induces reactive astrocytes and reduces perivascular flow and waste clearance.^9,78,79^ Sleep appears to influence amyloid-β accumulation via several mechanisms; amyloidogenesis appears to be increased via increased amyloid precursor protein (APP) cleavage as a result of sleep deprivation,^9,46,80^ while clearing via glymphatic pathways is simultaneously reduced.

Sleep deprivation leads to changes in AQP4 expression.^9,79^ In humans, some AQP4 single nucleotide polymorphisms (SNPs) correlated with worsened self-reported sleep quality and an increased amyloid-β burden.^56^ This suggests genetic variation in AQP4 can not only impact amyloid- β accumulation, but also sleep quality.

Stress caused by sleep deprivation might exacerbate the negative effects of disturbed sleep, as stress itself has been indicated as a risk factor for AD, PD and Huntington’s disease.^81–83^ Stress response also goes hand in hand with inflammation,^81^ decreased AQP4 expression and polarisation.^84^ Furthermore, chronic stress reduced CSF influx and increased Aβ_42_ build up in mice.^84,85^ Reduced brain clearance appears to be mediated via the glucocorticoid receptor (GR), as GR agonists result in reduction of glymphatic function, AQP4 expression and increased amyloid build-up, while anti-glucocorticoids rescue the phenotype and prevent glymphatic dysfunction.^84^ Disruption of glymphatic pathways might exacerbate the negative effect chronic stress has on the brain.^84,85^

Therefore, the glymphatic system could be of interest for future studies addressing the connection between various neurological problems that are observed as a result of sleep deprivation. The role that sleep plays that brain clearance further highlights its importance for combatting proteinopathies.

## Diet can support brain clearance by maintaining vascular health

Eating habits have a profound effect on health and can significantly increase or reduce the risk of developing a number of diseases. For instance, the ‘Mediterranean diet’ has been associated with reduced risk for developing AD,^86,87^ while high-glycaemic-index and high-fat diets are associated with increased amyloid accumulation and thus higher risk of developing AD.^88^ The effects that dietary choices have on neurodegeneration and brain health are complex, connected to nutrition-related changes in the function of multiple organ systems, and it often takes decades for effects to manifest.

Diet is the main cause of obesity, metabolic syndrome and type 2 diabetes mellitus (T2D), and these metabolic changes increase the risk for developing AD.^89^ Obesity and T2D are risk factors for hypertension and other cardiovascular morbidities.^90,91^ In humans, hypertension increases risk for dementia.^92–94^ Although the cause of hypertension varies between patients, the contributions of excess dietary sodium intake are widely recognised.^95^ Both T2D ^96^ and hypertension have been linked to decreased glymphatic function,^40^ thus offering one potential explanation for the relationship between T2D, hypertension and neurodegeneration. A study found that spontaneously hypertensive rats had higher CSF volume but decreased CSF influx, suggesting that perivascular CSF flow is compromised.^40^ The presence of tracer in the subarachnoid space surrounding the basal brain regions was the same in both hypertensive and normotensive groups, indicating comparable subarachnoid circulation, which suggests that the disruption of the CSF-ISF interchange is likely due to altered perivascular flow and clearance.^40^ An MRI study of patients with cerebral small vessel disease (SVD) found that decreases in cerebrovascular compliance and adaptivity lead to higher sheer forces generated by the pulse wave, while the total blood flow might actually be reduced due to the decreased compliance.^97^ Increases in pulsatility (difference in blood flow between systolic and diastolic phase) were also observed in SVD patients following intracerebral haemorrhage.^98^ This increase in vascular pulsatility led to decreased CSF pulsatility in the foramen magnum.^97^ Interestingly, many of these patients were also hypertensive, which may further contribute to decreased vascular compliance.^97^ Although not fully understood, this suggests that deviations from normal cerebrovascular function due to decreased compliance and subsequent high vascular pulsatility affect CSF circulation in both the ventricular system and the perivascular compartments. Conditions affecting vascular compliance, such as T2D or hypertension will thus likely exacerbate disturbances in CSF flow, which might lead to dysfunctional CSF-mediated brain clearance and in part explain the increased dementia risk associated with these conditions.

Polyunsaturated fatty acids (PUFAs), and particularly n-3 PUFAs (omega 3 fatty acids), are essential fatty acids that are important for maintaining vascular health. Central to this function appear to be their anti-hypertensive and anti-inflammatory properties, and their role in regulating platelet aggregation and blood lipid metabolism.^99^ High intake of docosahexaenoic acid (DHA), a type of omega 3 fatty acid, has been associated with lower risk of AD and PD, and interestingly post-mortem studies indicate deficiency of DHA in AD patients.^100^ PUFAs have recently been suggested to support glymphatic function as mice on a high PUFA diet showed increased CSF tracer influx and clearance, supporting the role of PUFAs in maintaining normal glymphatic function.^101,102^ Furthermore, transgenic mice expressing increased levels of omega 3 fatty acids in the brain showed protection against neuronal death from increased levels of amyloid beta.^101^ Interestingly, this protective effect was not seen in AQP4 knockout mice where the glymphatic system was inhibited.^101^

Alcohol use is a multifaceted public health issue; it increases the risk of various adverse health outcomes including several types of cancer and cardiovascular disease.^103^ However, at least some studies suggest that low consumption of alcohol leads to a decrease of all-cause mortality compared to zero consumption.^104,105^ This positive impact of low to moderate alcohol consumption on health may seem paradoxical, especially considering the detrimental effects of heavy drinking. Even one episode of binge drinking can result in adverse changes in the brain.^106^ Nevertheless, some studies suggest that light drinkers have reduced incidence of PD^107^ and dementia or AD^108,109^ when compared to non-drinkers, although not all studies show the same association. The effects of alcohol on the glymphatic system also show a J-shaped dose-response curve, where low levels seem to induce, and high levels inhibit the activity of the glymphatic system. When alcohol use is chronic, the glymphatic impairment is not rapidly reversed.^110^ Alcohol is a modulator of glymphatic function, resulting in an increased and decreased glymphatic function at low and high levels, respectively.^111^ The enhanced CSF-mediated clearance was attributed to nitric oxide (NO)-mediated dilation of blood vessels as an effect of low doses of alcohol.^112,113^ A role for vascular smooth muscle activity has also been implied as a mediator of the effect of alcohol on solute clearance.^112^ On the other hand, moderate levels of alcohol treatment in mice increased AQP4 levels but decreased polarisation, while low levels showed no difference compared to the control. Interestingly, based on the levels of glial fibrillary acidic protein (GFAP), reactive gliosis increased with binge-drinking levels of alcohol.^111^ Increased GFAP levels have also previously been connected to glymphatic dysfunction in mice^15,35^ and reported in AD patients.^114^ Furthermore, an EEG based sleep study on the effects of binge drinking in college students found that alcohol changed the EEG patterns to patterns associated with disrupted sleep.^115^ Thus, besides the cellular changes, alcohol may modulate glymphatic function by disrupting sleeping patterns.^115^ Thus, high levels of alcohol consumption have disruptive effects on glymphatic function mediated by both acute and chronic effects on various players in the glymphatic pathway, including AQP4 polarisation and disruption of sleep patterns. Low levels of alcohol on the other hand might have a beneficial effect on glymphatic function.

## Future perspectives

**Figure 4.**
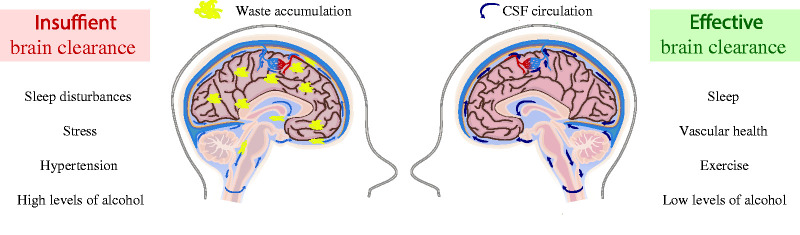
Lifestyle factors can modulate brain clearance and influence the risk of developing neurodegenerative diseases. Sleep disturbances, stress, hypertension and high levels of alcohol consumption lead to poor brain clearance and glymphatic dysfunction and may increase the risk for developing or exacerbating neurodegenerative diseases. On the other hand, factors that support normal brain clearance and function of the glymphatic system include adequate sleep (particularly deep phases of NREM sleep) and maintenance of vascular health through control of blood pressure, healthy dietary choices (e.g. diets high in Omega 3 PUFAs) and exercise. Low levels of alcohol consumption might promote brain clearance. Thus, lifestyle-related factors can modulate the risk for developing neurodegenerative diseases, potentially by enhancing or inhibiting brain clearance.

The glymphatic system has been indicated as a potential player that links CSF-mediated clearance to prevention of aggregated protein accumulation and thus decreased risk of neurodegenerative diseases. Many of the life style components that affect the glymphatic system also modulate the risk of neurodegenerative diseases (Figure 4). Dissecting the effects of healthy aging and possible risk factors of disease can help assess which lifestyle factors form a major risk for neurodegeneration, and on the other hand might elucidate mechanisms to be targeted as new therapies. However, in order to fully translate the findings of the glymphatic pathways discussed in this review, it is vital to understand whether the physiology of the glymphatic system is conserved across species. The majority of the studies on the glymphatic system have been done in rodents, although some human studies support the existence of glymphatic pathways.^116–119^ Similar to glymphatic animal studies, it is possible to study the distribution of contrast agent injected into the CSF in humans.^116,120^ Assessment of perivascular contrast agent enhancement makes it possible to study CSF distribution in the key anatomical compartments of glymphatic function, and support the persistence of the glymphatic system in humans.^119^ MRI measurements suggest that CSF contrast agent enters the brain and is subsequently cleared, and the clearance process is delayed in patients with dementia.^116^ Measurement of apparent diffusion coefficients using diffusion tensor imaging (DTI) can be used to study mechanisms of fluid transport in the brain, and help to establish whether fluid movement can be explained by diffusion or if bulk flow is likely to form a part of the flow.^118^ As clearance rates appear reduced in patients with dementia,^116^ measuring clearance might have predictive value in clinical settings.

Use of contrast agents injected into the CSF is required to enhance visibility of perivascular or intraparenchymal CSF flow to measure it using MRI. Lumbar injections of gadolinium-based contrast agents to assess CSF leakage in patients is already in use.^117^ However, the invasive nature of such methods involving gadolinium-based contrast agents for experimentally assessing glymphatic function makes it largely unsuitable to directly replicate rodent studies in healthy volunteers. To avoid the use of contrast agents, macroscopic CSF flow can be measured non-invasively in the ventricular system, for example in the cerebral aqueduct^27^ or the foramen magnum.^97^ One study found CSF pulsatility to be increased in MCI and even more so in normal pressure hydrocephalus.^19^ However, in AD patients the pulsatility had returned to normal range. This suggests that CSF pulsatility might be useful for differential diagnosis of AD or normal pressure hydrocephalus. Furthermore, longitudinal studies may offer predictive value of MCI progression to AD.

Development of new imaging techniques for studying CSF flow and homeostasis is vital for better understanding the CSF-mediated brain clearance in humans. As a step towards such an approach, we showed how ultra-high field 7 T MRI can be used to quantify flow of CSF in the cerebral aqueduct and its correlation with the heart cycle in humans, as an attempt to bridge the translational gap between animal studies and clinical observations. Further studies, particularly in higher mammals and humans, are needed to assess whether glymphatic dysfunction significantly contributes to the progression of neurodegenerative disease. Another important question is whether it is possible to prevent or alleviate neurodegenerative diseases by further enhancing brain clearance via therapies targeting glymphatic pathways.
